# Jail-based interventions to reduce risk for opioid-related overdose deaths: Examples of implementation within Ohio counties participating in the HEALing Communities Study

**DOI:** 10.1186/s40352-024-00307-3

**Published:** 2024-12-05

**Authors:** Joel Sprunger, Jennifer Brown, Sofia Rubi, Joan Papp, Michael Lyons, T. John Winhusen

**Affiliations:** 1 Addiction Sciences Division, Department of Psychiatry & Behavioral Neuroscience, University of Cincinnati College of Medicine, Cincinnati, Ohio, USA; 2 University of Cincinnati Center for Addiction Research, Cincinnati, Ohio, USA; 3 Department of Psychological Sciences, Purdue University West Lafayette, West Lafayette, USA; 4 Department of Emergency Medicine, MetroHealth, Cleveland, USA; 5 Department of Emergency Medicine, The Ohio State University, Columbus, USA

**Keywords:** Jail, Overdose, Opioid use disorder, Naloxone, Buprenorphine, Naltrexone, Peer support

## Abstract

**Background:**

Opioid-related overdose is a leading cause of death for criminal legal-involved individuals and, although naloxone distribution and medications for opioid use disorder (MOUD) are effective means for reducing post-release overdose death risk, jail-based availability is limited. This case report describes the challenges faced by three Ohio communities as they implemented evidence-based practices (EBPs) in jails to combat post-release opioid overdose deaths.

**Method:**

We present case examples of how barriers were overcome to implement jail-based EBPs in three Ohio communities (two urban and one rural) as part of the HEALing Communities Study (UM1DA049417; ClinicalTrials.gov Identifier: NCT04111939). Of the 18 participating Ohio HEALing Communities Study counties, we highlight 3 communities for the novelty of their EBPs implemented, the challenges that they faced, and their rural/urban status. We present descriptive data regarding the EBPs that they implemented and discuss the challenges identified by HEALing Communities Study staff with first-hand experience facilitating their implementation.

**Results:**

Newly implemented interventions included overdose education and direct provision of naloxone to incarcerated individuals upon release (2 of 3 communities), initiating MOUD prior to release (3 of 3), linkage to ongoing MOUD treatment in the community (2 of 3), peer support-facilitated treatment retention efforts (2 of 3) and emergency housing (1 of 3) in the immediate post-incarceration period. Common challenges that emerged included skepticism about the need and feasibility of implementing EBPs to reduce overdose and death, lack of knowledge about the options available and whether external agencies may assist, and difficulty engaging stakeholders to overcome inertia.

**Conclusions:**

Creative flexibility, calm persistence, technical facilitation, and collaboration with community service providers were assets that helped these Ohio jails implement evidence-based strategies that combat the opioid epidemic and reduce the likelihood of post-incarceration overdose and death in a high risk, formerly incarcerated population.

## Background

Approximately 70% of people who use illicit opioids, and 15–20% of individuals with opioid use disorder (OUD), have been incarcerated or involved with the criminal legal system in some way and are at a higher risk for overdose mortality (Krawczyk et al., 2022; Lim et al., 2023; Macmadu et al., 2020). The leading cause of death for criminal legal system-involved individuals is overdose, and the majority are opioid-related (Showalter et al., 2021). Individuals with OUD or a history of opioid use are at heightened risk of experiencing opioid-related overdose following their release from jail or prison, especially in the first two weeks (Lim et al., 2023; O’Connor et al., 2022). This is due to a number of factors, including a reduced tolerance for opioids from enforced abstinence, and the rise of illicit fentanyl—a high potency opioid often mixed with other illicit substances (Joudrey et al., 2019). Thus, efforts to initiate treatment for OUD, provide naloxone—an opioid antagonist medication that rapidly reverses opioid overdoses—and develop a post release treatment plan are all important for safely navigating this dangerous transition.

OUD screening in jails, coupled with treatment, substantially decreases overdose following release (Macmadu et al., 2020). Methadone, buprenorphine, and naltrexone are the three FDA-approved medications for opioid use disorder (MOUD). Methadone and buprenorphine have the strongest evidence demonstrating their effectiveness for reducing overdose incidence, with weaker evidence supporting naltrexone (Larochelle et al., 2018; Santo et al., 2021). Simulation modeling using 2016 data from the Bureau of Justice predicted that 1,840 lives could have been saved in the US that year if any MOUD had been provided in jails and prisons; an additional 4,400 lives would have been saved if individuals who screened positive for OUD were offered MOUD treatment following their release (Macmadu et al., 2020).

Despite their efficacy, jail-based MOUD and opioid overdose education and naloxone distribution (OEND) access is limited in the United States (Macmadu et al., 2020). A 2023 bulletin by the U.S. Department of Justice reported that only 24% of local jails continued MOUD and 19% initiated MOUD upon entering incarceration and only 25% of jails provided naloxone at release (Maruschak et al., 2023). Further, existing jail-based OUD treatment models are not standardized and vary drastically across states and systems. For example, it is not uncommon that individuals taking MOUD prior to incarceration are required to taper off and go through withdrawal upon entering jail (Grella et al., 2020). In addition to these hurdles, other considerable barriers to providing OEND and MOUD exist, including short jail stays that limit opportunity to initiate treatment, stigma, funding for treatment staff and pharmaceuticals, and poor access to community resources to support recovery (Krawczyk et al., 2022).

Jail-based OEND efforts are also efficacious in preventing opioid-related overdose fatalities by increasing the knowledge and availability of the overdose reversal medication (Cherian et al., 2024; Lim et al., 2023; Macmadu et al., 2020). Trained OEND professionals provide overdose rescue training including education on overdose prevention, recognition, and proper administration of naloxone. These programs also educate participants on existing laws that promote overdose reporting by removing the threat of legal consequences (Huxley-Reicher et al., 2018). A study conducted in the Los Angeles County jail revealed that up to 40% of individuals who are incarcerated expressed an interest in overdose prevention and response training, including individuals without a history of opioid use or overdose (Davidson et al., 2019).

The current case report describes the major challenges and successes of implementing evidence-based practices (EBPs) for reducing opioid-related overdose and fatalities within Ohio jails as part of the Helping to End Addiction Long-term^®^ (HEALing) Communities Study (HCS). Specifically, these practices include strategies that actively educate and place naloxone kits in the hands of high-risk individuals (i.e., active OEND rather than relying on referral or self-request), add or expand the availability of MOUD treatment for individuals with OUD, link high-risk individuals to MOUD treatment, and improve engagement and retention with MOUD treatment. Using illustrative examples from urban and rural Ohio counties, we highlight the barriers encountered and outline the effective methods we employed to navigate them, enabling the timely implementation of these programs.

## Method

### Participants

The three communities that we highlight were three Ohio counties participating in the HEALing Communities Study (HCS). These communities were chosen as case examples for the current report because of (a) novelty in terms of their implemented EBPs, (b) the challenges that they faced and how they were overcome, and (c) their rural/urban status (i.e., two representing large urban counties; one representing a smaller rural county). To preserve their anonymity, these communities will not be named. County 1 is a large urban community (rural population = 2.4%) with a population of over 800,000 residents. County 2 is another large urban community (rural population = 1.4%) with a population of over 1 million residents. County 3 is a rural community (rural population = 71.7%) with a population of over 50,000 residents.

#### HEALing communities study trial design

The HEALing Communities Study was a parallel-group, cluster randomized, unblinded, wait-list controlled trial of 67 communities in four states—Kentucky (*n* = 16), Massachusetts (*n* = 16), New York (*n* = 16), and Ohio (*n* = 19)—with rural communities representing at least 30% of those within each state. In HCS, communities were the unit of analysis and consisted of counties (*n* = 48 in OH and KY) or cities/towns (*n* = 19; 16 in MA and 3 in NY). Communities highly impacted by opioid-related overdose fatalities (i.e., a rate of ≥ 25 opioid-related fatalities per 100,000 people, based on 2016 data; Walsh et al., 2020) were recruited to participate.

#### HCS study procedures and intervention

Prior work describes the conceptualization of HCS (Chandler et al., 2020; El-Bassel et al., 2020), the study protocol (Walsh et al., 2020), the Communities That Heal (CTH) intervention (Sprague Martinez et al., 2020), and its overall effects (The HEALing Communities Study Consortium, 2024). Briefly, community coalitions were formed or adapted from existing coalitions. Based on a data-informed needs assessment of their community, coalitions selected strategies (Young et al., 2022) to implement from the Opioid-overdose Reduction Continuum of Care Approach (ORCCA; Winhusen et al., 2020), a compendium of EBPs that reduce opioid overdose mortality. Communities were required to implement at least one strategy from each of five menu categories of EBPs: (a) active OEND; (b) expanding access to MOUD; (c) expanding linkage to MOUD services; (d) supporting MOUD treatment engagement and retention; and (e) safer opioid prescribing and dispensing practices. In HCS, MOUD EBP strategies focused specifically on expanding access to buprenorphine and methadone due to their stronger evidence base. Each community was allocated an equal amount of funding to be spent toward EBP implementation across healthcare, behavioral health, and criminal legal settings; how those funds were spent was determined by the community coalitions.

#### Technical facilitation to support EBP implementation in the Ohio site

Within Ohio, each of the 18 participating counties had a full-time Intervention Facilitator who was dedicated to managing and supporting all EBP implementation efforts in healthcare, behavioral health, and criminal legal sectors across the county. Additionally, six Intervention Design Teams (IDTs) were created, each focused on the setting where the EBP was to be developed and implemented. Separate IDTs oversaw the following settings: (i) substance use disorder and behavioral health treatment, (ii) criminal legal system, (iii) public health and community-based organizations, (iv) emergency medical services, (v) emergency departments, and (vi) pharmacies. Each IDT was comprised of Technical Facilitators, or experts experienced with EBP implementation within the setting. Technical Facilitators assisted in the development of specific EBP implementation plans and provided ongoing technical consultation to support EBP implementation. This model allowed for peer-to-peer technical facilitation to support service agency partners’ EBP implementation efforts. Technical Facilitators from Ohio’s Criminal Legal System IDT included emergency medical physicians, behavioral health professionals, and members of law enforcement.

#### Institutional review & data safety and monitoring boards

In accordance with the Declaration of Helsinki standard, the protocol (Pro00038088) was approved by Advarra Inc., the HCS single Institutional Review Board (sIRB), and was granted a Waiver of Consent and a Full Waiver of HIPAA Authorization for secondary data analysis (3/6/2023, MOD00521925). The Data Safety Monitoring Board (DSMB), chartered by the National Institute on Drug Abuse (NIDA), was an independent group charged with monitoring the safety of participating communities.

#### Identifying challenges and lessons learned

The list of jail-based OEND and MOUD EBP implementation challenges and lessons learned was synthesized from review of (a) the minutes from relevant IDT (*n* = 59 for Counties 1 & 3 from 05/2020 to 5/2022; *n* = 29 for County 2 from 10/2022 to 11/2023) and stakeholder meetings for jail-based EBPs (County 1, *n* = 10 from 5/2020 to 4/2022; County 2, *n* = 12 from 11/2022 to 11/2023; County 3, *n* = 9 from 9/2020 to 5/2021), (b) implementation plans detailing the role of all parties in executing each EBP in each jail (County 1, *n* = 7 implementation plans; County 2, *n* = 3; County 3, *n* = 3), and (c) personal communication with HCS Intervention Facilitators and Technical Facilitators directly involved with their implementation. The first author devised the initial list based on the review of materials above and the writing team (which includes members of the HCS Criminal Legal System IDT) agreed that it captured a representative sample of challenges faced by Ohio jails implementing these EBPs.

## Results

### Evidence based practices

Table 1 presents the community characteristics, pre-HCS existing practices, and EBPs implemented by our three sample Ohio counties. As described above, HCS required communities to select EBPs from five ORCCA menu categories but only the first four categories (i.e., active OEND, adding/expanding MOUD, linkage to MOUD, and engagement/retention in MOUD) were relevant for the present paper. Here, we describe the strategies that our three sample counties implemented by EBP category. All jail-based EBP strategies selected by these three counties were successfully implemented.

**Table 1:** Community characteristics, existing practices, and evidence-based practices implemented by three Ohio county jails participating in the HEALing Community Study

	County 1	County 2	County 3
Urban/rural	Urban	Urban	Rural
Population	> 800 000	> 1 million	> 50 000
Existing opioid overdose education and naloxone distribution (OEND) services	None	Naloxone kits upon release	None
Existing medication for opioid use disorder (MOUD) services	Continue MOUD for those receiving it pre-jail	Injectable naltrexone for inmates with OUD	None
Implemented OEND strategies	Four jail-based OEND strategies	None	One jail-based OEND strategy
Implemented MOUD strategies	• Buprenorphine for individuals who are incarcerated and have OUD • Peer-based linkage to post-release MOUD	• Buprenorphine (injectable) for individuals who are incarcerated and have OUD • Peer-based linkage to post-release MOUD • Peer support and contingency management to increase MOUD retention	• Continue MOUD for individuals who are incarcerated who had been receiving it pre-jail • Buprenorphine or injectable naltrexone for individuals who are incarcerated and have OUD* • Housing support to improve MOUD retention

*Post-release treatment continued with the same provider

#### Active opioid overdose education and naloxone distribution (OEND)

Two of the three counties selected and implemented active OEND strategies, but their scope and quantity of strategies differed greatly. For example, County 1 implemented four separate strategies for jail-based OEND, including the provision of naloxone education and kit distribution for pretrial individuals incarcerated short-term and released on bond (~$20,000 HCS funds for staff training and salary; naloxone kits provided by public health department), post-trial individuals being released after lengthy periods of incarceration ($0 HCS funds; naloxone provided by public health department and staffing provided by non-profit organization), and post-trial individuals involved in a jail-affiliated residential treatment program ($0 HCS funds; naloxone provided by public health department and staffing provided by host agency). Further, County 1 implemented a program to provide naloxone kits with belongings for pre- and post-trial individuals so that naloxone was available immediately upon release ($0 HCS funds; naloxone provided by public health department). County 3 chose to provide overdose education and naloxone training via an educational video then, upon release, pre- and post-trial incarcerated individuals received a “go bag” with naloxone kits, educational pamphlets, and referral information (~$40,000 HCS funds; covered staff effort, technology, naloxone, and distribution materials). County 2 already had a program that provided naloxone kits upon release and did not implement a new jail-based active OEND strategy with HCS.

#### Adding or expanding availability of medications for opioid use disorder

All three counties selected and implemented strategies that added and/or expanded MOUD availability within the jail for incarcerated individuals. County 1 had an existing policy to continue medications for pre- and post-trial incarcerated individuals already inducted on MOUD prior to entry. However, they chose to expand their service to include buprenorphine induction for interested post-trial inmates with OUD; pre-trial inmates were eligible as long as it was feasible within their variable incarceration period. The county negotiated with the jail’s corporate medical provider to implement necessary procedures to determine eligibility for MOUD induction including medical exams and diagnosis, informed consent, medication administration and monitoring, as well as coordination between jail medical and peer recovery support staff for linkage to post-release MOUD care (described below). County 1’s coalition allocated approximately $40,000 of their HCS funds for technical facilitation to establish the necessary infrastructure to support a buprenorphine induction and maintenance program within the jail. Once established, sustainability plans to continue the program included budgetary modifications for the department by the county, securing state resources for medication reimbursement, grants from the county mental health and recovery services board, and other grant funding opportunities.

County 2 had an existing program that inducted interested individuals on injectable extended-release naltrexone during their incarceration along with subsequent referral to continue MOUD care in the community post-release. However, they chose to expand their MOUD programming by implementing a pilot program to offer inductions on injectable extended-release buprenorphine along with peer recovery support-facilitated linkage to ongoing MOUD care in the community following their release (described below). These services were available to eligible post-trial inmates as well as pre-trial inmates with sufficient incarceration periods to complete the MOUD induction procedures. Implementation of this program did not require the county to spend HCS funds, as the county board decided to commit support for the pilot program outright.

County 3’s jail lacked MOUD programming prior to HCS involvement and this presented an opportunity for significant expansion. They chose to implement programming to address two needs. First, they added the ability to continue existing MOUD for incoming pre- and post-trial individuals already taking MOUD. Second, they implemented a program to conduct screening and diagnostic assessments and induct eligible pre- and post-trial individuals on buprenorphine or extended-release injectable naltrexone. County 3 partnered with a local healthcare provider to provide these services within the jail and utilized telemedicine for weekly follow-up visits with MOUD providers. Also, individuals receiving MOUD could continue their treatment relationship with their MOUD provider post-incarceration through the same local agency. These efforts allocated approximately $20,000 of the county’s funds to establish the infrastructure for induction and maintenance of MOUD care and the medications themselves. The local partner healthcare provider will sustain the program after HCS funds are expended.

#### Linkage to medications for opioid use disorder

Counties 1 and 2 piloted EBP strategies to link pre- and post-trial incarcerated individuals to MOUD services post-release. In both cases, the jails contracted with peer recovery specialists to build relationships with individuals receiving MOUD treatment during their incarceration and connect (or, reconnected for individuals taking MOUD prior to incarceration) them with ongoing care in the community prior to their release. Locating an MOUD treatment program post-release is a common challenge noted by people who were formerly incarcerated (Treitler et al., 2022). For all inmates receiving MOUD, the peer support specialists overcame this barrier and facilitated their continuity of care by scheduling intake appointments with community MOUD providers. Additionally, they assisted with transportation to appointments and connected released individuals with social support resources in the community. For pre-trial individuals with shorter incarceration periods, the peer support specialists met them in the release area to help arrange intake appointments with community MOUD providers and attempted to reduce attendance barriers.

County 1’s coalition allotted approximately $180,000 to support their MOUD linkage strategy. Associated costs included technical facilitation, durable infrastructure, and the salaries of a peer recovery chemical dependency counseling assistant and two part-time licensed independent clinical social workers. Sustainability plans included funding from the local mental health and recovery services board, partnerships with other agencies, and external public and/or foundation funding. County 2 allotted $80,000 of their HCS funds toward their MOUD linkage strategy to support training costs and protocol development, technology, and salaries for two part-time peer supporters and one recovery support professional. Sustainability plans includde funding from the county administrative board, partnerships with local agencies, grant applications, and foundation support. Although County 3 did not specifically select a jail-based MOUD linkage strategy as part of HCS, their partnership with a local treatment provider to administer their MOUD strategy above effectively linked individuals to MOUD post-release by allowing them to continue with the same agency once they re-entered the community.

#### Medications for opioid use disorder engagement and retention

Counties 2 and 3 implemented EBP strategies aimed at engaging and retaining people who were formerly incarcerated in MOUD care post-release. However, they approached this goal from different angles. County 2 utilized the peer recovery specialists hired to support their linkage strategy described above to provide ongoing one-to-one retention support that facilitated engagement with community MOUD providers post-release. Further, their peer supporters maintained contact, monitored MOUD compliance, and applied contingency management to reinforce markers of MOUD engagement, compliance, and retention as individuals who were formerly incarcerated established with their community-based treatment providers. Target behaviors included MOUD appointment attendance and medication compliance. Motivational incentives for contingency management consisted of gift cards for items necessary to support re-entry into the community, including hygiene items and clothing. In addition to the funds expended toward their linkage strategy described above, County 1 allocated approximately $10,000 toward their contingency management pilot program. This covered the costs of signage to raise program awareness, treatment education materials, and the contingency management reward gift cards (~$50 for 20 individuals per month for 8 months). Sustainability plans included possible funding through third-party payors, the local mental health services board, the United Way, and relevant county government departments.

County 3 implemented a novel strategy to address the housing instability of people who were formerly incarcerated seeking MOUD post-release. Recognizing that housing instability affects the likelihood of MOUD engagement (Johnson & Fendrich, 2007), County 3 provided housing in a hotel for up to one month for post-trail jail-involved individuals. This provided a foundation as these individuals established MOUD care with community providers and sought employment. In addition to housing support, jail staff provided beneficiaries of this strategy also received educational and community resource information tailored for individuals with OUD prior to release. County 3 allocated approximately $25,000 of their HCS funds to support technical assistance to develop the protocols, print educational materials about available local barrier reduction services, staff effort for case management during their stay, and hotel fees. Sustainability plans for this strategy included ongoing price negotiation with the hotel, local partnerships, and foundation funding.

## Discussion

### Challenges and lessons learned

The CTH intervention involved ongoing collaboration between community coalitions and technical experts to implement EBPs for reducing opioid-related overdoses and fatalities. These partnerships combined the expertise of local coalitions who know their community best with expert consultants providing first-hand experience implementing these practices in similar settings. As with any change in practice or policy, challenges emerge that can stifle progress or discontinue a project entirely. In this section, we discuss key challenges faced by our communities standing up the jail-based EBPs described above, and present lessons learned throughout their successful implementation. We begin with common challenges observed across communities and conclude with specific examples that illustrate flexible implementation of OEND and MOUD strategies. It is important to note that although we chose to categorize these challenges as presented in our experience, we recognize that these categories are not mutually exclusive, and that issues like stigma can apply across multiple domains.

#### Skepticism about EBP need and feasibility

In our experience with participating communities, skepticism regarding what strategies may be implemented in a timely manner, and whether they would be worth the investment, was common during the EBP selection phase. In the case of County 1, skepticism was expressed by the coalition with representation from the jail regarding a possible jail-based OEND strategy due to a past experience; a volunteer organization attempted to distribute naloxone outside of the jail, but the effort ultimately failed due to disorganization. County 3’s coalition, on the other hand, was skeptical that new OEND and MOUD efforts were necessary, or even possible, given the smaller population and resource limitations of the rural county jail. All communities were concerned about the assumed likelihood that some forms of MOUD (e.g., sublingual buprenorphine) may be diverted. An injectable formulation was favored because of the reduced risk of diverting the medication, whether by choice or by coercion. Prior to HCS, the most commonly available injectable MOUD formulation was for extended-release naltrexone. However, the literature supported buprenorphine and methadone as superior, with benefits including a lower barrier to induction (e.g., opioid abstinence window of around 24 h for buprenorphine; up to 7 days for naltrexone). Even so, correctional facilities were reluctant to consider sublingual buprenorphine despite its low barrier to entry, higher treatment retention, and success lowering risk for overdose fatality (Lee et al., 2018; Nunn et al., 2009; Paul et al., 2023). To address these concerns, we found it helpful to have consistent contact and regular meetings (face-to-face and virtual) with relevant decision makers and stakeholders to discuss these points, find common ground in the good work being done currently and recognize areas to improve, respectfully present evidence supporting these practices, and establish feasibility via testimonials from technical experts or representatives from peer counties that had successfully implemented similar strategies. In other counties not highlighted here, we found that starting with injectable formulations was a helpful launching point for considering other formulations in the jail setting.

#### Political issues and competing interests

##### What is on the table?

Stigma was a common issue that communities encountered when considering options for EBPs. Stigma regarding OEND (e.g., “that naloxone administration only enables addiction”) and MOUD (e.g., that it is “only trading one pill for another”) was a considerable barrier that needed to be addressed in communities, generally. As above, consistent meetings, respectful listening, and relationship building were critical to ensure that concerns were heard, shared goals were elicited, and common ground was established as a foundation to move forward collaboratively. As trust developed, presentations from individuals with lived experience with addiction and, when possible, a particular proposed intervention (e.g., individuals with a use disorder who benefitted from MOUD; parents/friends who lost a loved one to overdose) helped humanize the potential impact of piloting the strategy. Additionally, we found that enlisting trusted experts as champions of the intervention (e.g., buprenorphine induction in the jails) from the local or neighboring counties was effective at increasing willingness to move forward. Finally, our technical facilitation model permitted peer-to-peer conversation and support so that concerns raised by law enforcement personnel were addressed directly with experienced HCS-affiliated law enforcement personnel (e.g., sheriff to sheriff). Together, we found that these approaches helped to challenge the stigma held by decision-makers and agencies while increasing willingness to pilot EBP programs in the jails that address the high-risk post-release period. In our experience, community coalitions and jails were relatively more receptive to OEND, and we found that once an OEND strategy had been implemented, initially hesitant stakeholders were more open to considering MOUD options. However, in some cases, the barriers were only resolved by turnover in personnel or replacement by newly elected officials.

##### Who should be at the table?

A common challenge that emerged across communities was determining who should be involved with EBP implementation planning; a process led by local coalitions taking the preferences of the jails into consideration. Ultimately, coalitions selected the EBP strategies for their jails as well as the agencies that would implement them. Jail administration and sheriff department representation within coalitions was generally greater in urban counties than rural counties, and that helped bridge the gap between EBP selection and jail engagement for implementation. For rural counties with little to no coalition representation, early involvement of jail administration in the EBP selection process was important to align goals and ensure a feasible implementation pathway. Often, jail preferences included contracting with external agencies to provide a new service instead of creating new in-house positions. In the larger urban counties with many potential service agencies, it was necessary to fairly narrow the pool and determine the best candidates for the contract. In each case, this generally involved identifying which agencies had the licensure and capacity to deploy the service in the jail in a timely manner and at a reasonable price. These determinations were made through regular discussions with decision-makers about the pool of options and inviting candidates to these meetings to address questions and concerns.

##### What if they cannot come to the table?

Across counties, situations emerged in which we had difficulty engaging key stakeholders and decision makers in the implementation process. One example was a sheriff overseeing the construction and hiring of staff for a new correctional facility while managing responsibilities for the existing jail during the COVID-19 pandemic. Understandably, it was difficult to engage the sheriff to move forward with implementing new policies and practices in the context of their many other pressing responsibilities. We resolved this issue by maintaining regular communication with the sheriff’s command staff and facilitating engagement with other county offices necessary to make progress on implementation so that we only solicited the sheriff’s direct involvement when necessary.

#### Challenges for jail-based OEND

County 1 required a flexible approach to jail-based OEND, with several adjustments along the way to overcome obstacles that emerged during implementation. Early in the implementation planning phase, the sheriff was hesitant to have personnel conducting the naloxone training and distributing kits in the jail’s release area. Additionally, the sheriff was wary about an alternative strategy in which naloxone kits would be included in an individual’s belongings when they are released. This alternative was proposed because leave behind programs have been shown to effectively increase the availability of naloxone and, in turn, combat the number of opioid-related deaths (Walley et al., 2013). However, the sheriff was concerned about whether individuals being released would receive sufficient training for what was then a prescribed medication. For context, recent revision of Ohio state law in 2021 allows pharmacists, pharmacy interns, and healthcare providers to provide naloxone to individuals upon release from hospitals and correctional facilities without a formal prescription (Ohio Board of Pharmacy, 2021). Statewide health initiatives including Project Deaths Avoided with Naloxone (DAWN) collaborate with jails to provide naloxone kits and training on proper administration (Ohio Department of Health, n.d.). Although studies conducted in Massachusetts found no significant difference in successful naloxone administration among trained and untrained individuals (Doe-Simkins et al., 2014), the sheriff was open to an initial strategy that involved a trained peer support specialist waiting outside of the jail to engage discharged individuals and provide the naloxone training and kits. An issue that emerged, however, was that few individuals were engaging with the peer support specialist as they were understandably eager to move on. We brought this observation to the newly elected sheriff and they decided to move the OEND program inside the release area, as initially proposed.

The county was motivated to schedule their peer support specialist so that they were available during peak release times to distribute as many naloxone kits as possible. In practice, peak release times were difficult to predict, and this resulted in downtime for the peer support specialist that could be better spent on other activities. To address this, the sheriff approved the alternative strategy proposed above; the peer support specialist trained soon-to-be discharged individuals prior to their release who then received a kit with their belongings. The sheriff approved a proposal to have the peer support specialist dedicate non-peak time to engagement, naloxone training, and kit distribution at local overdose hotspots near the jail.

#### Challenges for jail-based MOUD

Counties 1 and 2 required contract renegotiation with their corporate corrections medical providers before they could implement their expanded MOUD strategies. While this process progressed relatively quickly for County 1, County 2 experienced a slower process. In addition to the sheriff’s limited availability described above, the external medical provider was difficult to engage to discuss the proposed injectable buprenorphine induction and maintenance strategy. Although staff at various levels attempted emails and phone calls to engage them, the breakthrough came with peer-to-peer conversations between an Ohio-based expert technical advisor and the provider’s medical director. Through these relationship-building discussions, it became known that the hesitancy to engage was due to confusion about implementation roles for the new strategy. When it was made clear that the medical provider would be responsible for performing the services in the updated contract, their engagement improved, and implementation moved forward at a more rapid pace.

County 3 was unsure which MOUD options would be feasible given their jail’s occupancy and resources. We collaborated with the sheriff’s command staff to brainstorm workable solutions that may fit their needs, including their limited space, staffing, and funding. Rather than attempting to hire staff in-house, which would require precious time for recruitment, training, and space, we agreed that locating an external provider would be the most expedient solution. Rather than pursuing a contract with a corporate corrections medical provider, the sheriff’s office ultimately decided to partner with a local treatment provider. By enlisting a local treatment agency to enter the jail to conduct assessments, administer medications, and conduct weekly follow up appointments via telehealth, this partnership allowed many benefits. These include side-stepping many logistical issues (e.g., hiring and housing staff, storing, administering, and monitoring MOUD) and supporting a natural continuity of care (i.e., the ability to follow up with the same MOUD treatment provider post-release).

### Limitations

One notable limitation of the study is the lack of data regarding the number of individuals served by the programs implemented by our case example counties. The Ohio site’s focus in HCS was on outcomes at the level of implementation (e.g., which EBPs were selected and implemented in sectors within participating communities). As such, our agreements with participating agencies did not include data collection at the level of service delivery (i.e., number of kits distributed to distinct individuals, number of individuals inducted/maintained on buprenorphine, number of individuals linked to MOUD post-release). Although these data would be valuable, we lack the ability to collect and present these data accurately across our participating Ohio communities given the burden it would place on agency staff. Future research will benefit from capturing data at multiple levels to show not only which EBPs were implemented, but their reach within the communities. Even so, we believe that our lessons learned are valuable to communities of similar sizes and resources considering OEND and MOUD EBP implementation within their jails.

## Conclusions

The list of potential barriers to implementing jail-based strategies for OEND and MOUD is daunting and differs from jail to jail. Even so, our case examples representing diversity in community size and resources illustrate that collaboration among (a) experts who know their communities best and (b) those with technical expertise in jail-based settings makes for a powerful combination that results in programs connecting individuals at high risk for overdose and death with life-saving medications through naloxone kits upon release, starting and continuing MOUD during incarceration, and ensuring their continued OUD treatment in the community post-release. Calm persistence and creative use of available resources helped to establish programs and referral pipelines in communities that once struggled to see the need or a way forward. The challenges faced by these communities are not unique to Ohio and we hope our lessons learned serve as a guide for other communities seeking to implement EBPs of their own.

### Experience-based tips for implementing jail-based OEND and MOUD EBPs

- Calmly persist in the face of setbacks as priorities and administrations shift. Engage with those who will listen and take advantage of opportunities to develop relationships. We found that meeting in person was sometimes valued more than by teleconference.
- Humanize the intervention with testimonials by persons with lived experience. Enlist local experts to champion these efforts. Encourage peer-to-peer discussions to address stigma and provide technical expertise to overcome inertia and disbelief that an intervention is possible within in a jail setting.
- Consider framing a new EBP as a “pilot” or “experimental program” that will be evaluated for its efficacy. This may help reduce the sense of commitment and ownership by decision-makers who may be skeptical of a new service while providing a pathway to demonstrate its value.
- Beginning with a focus on OEND can eventually increase receptiveness to MOUD options.
- Starting with injectable formulations of MOUD can help overcome concerns about diversion and eventually lead to consideration of other formulations in the jail setting.
- Pairing naloxone kits in personal property boxes with overdose education and naloxone training prior to release can improve OEND dissemination and distribution more so than attempting to engage individuals upon release.
- Develop local interagency relationships to provide services in the jail and allow for natural continuity of care by following up with the same community provider post-release.
- To the extent possible, utilize available state and local resources for sustainable programs (e.g., state-supplied naloxone; local medical and behavioral health providers interested in developing referral pipelines, state/federal opioid use disorder treatment-related initiatives, opioid settlement funds/foundations).
- Utilize peer support to develop relationships during incarceration that will continue post-release. Peer supporters empowered with contingency management can reduce re-entry barriers (e.g., by making intake appointments, arranging transportation), play the critical role of a recovery-promoting source of emotional support (because prior support networks are often disrupted), and encourage the development of a recovery-consistent lifestyle by reinforcing post-release MOUD engagement and medication compliance through motivational incentives.

## Data Availability

The qualitative data evaluated for this case report are not publicly available because they consist of meeting minutes and implementation plans with named agencies partnering with the HEALing Communities Study research team.

## Abbreviations

CTH: Communities that HEAL
EBP: Evidence–based practice
HCS: HEALing Communities Study
HEAL: Helping to End Addiction Long–term^®^ (HEALing)
MOUD: Medications for opioid use disorder
OEND: Opioid overdose education and naloxone distribution
ORCCA: Opioid–overdose Reduction Continuum of Care Approach
OUD: Opioid use disorder
